# Biomarkers in inflammatory bowel diseases: insight into diagnosis, prognosis and treatment 

**Published:** 2017

**Authors:** Mohsen Norouzinia, Vahid Chaleshi, Amir Houshang Mohammad Alizadeh, Mohammad Reza Zali

**Affiliations:** 1 *Gastroenterology and Liver Diseases Research Center, Research Institute for Gastroenterology and Liver Diseases, Shahid Beheshti University of Medical Sciences, Tehran, Iran *; 2 *Basic and Molecular Epidemiology of Gastrointestinal Disorders Research Center, Research Institute for Gastroenterology and Liver Diseases, Shahid Beheshti University of Medical Sciences, Tehran, Iran*

**Keywords:** Inflammatory bowel diseases, Cytokines, Biomarkers

## Abstract

Inflammatory bowel disease (IBD) is a chronic disease of unknown etiology which mostly involves the intestine and requires a personalized approach for treatment. IBD represents a heterogeneous group of patients with inherently variable disease courses. Hence, the heterogeneity of patient populations may delay the diagnosis, clinical practice and initiation of appropriate treatment. Use of biomarkers for diagnosis and management of IBD is still necessary. Descriptions of the immunological pathway abnormalities in IBD improve assessment to identify the patient’s disease status, and relative risk of progression to complicated disease behaviors, and this information may ultimately influence therapeutic decisions. In this study, we try to explain the role of biomarkers in early diagnosis, estimating prognosis, and target agents for correct managements of IBD’s patients. This information might be important to provide insight into emerging panels of multiple IBD biomarkers and highlighting the essential role of personalizes panel for each patient.

## Introduction

 Inflammatory bowel disease (IBD), including two subtype, ulcerative colitis (UC) and Crohn’s disease (CD), is a chronic inflammatory disorder of unknown etiology which includes two subtypes – ulcerative colitis (UC) and Crohn’s disease (CD). Immunological, genetic and environmental factors have been involved in the progression of chronic inflammation of intestine (1, 2). The diagnostic and prognostic features of IBD are usually based on a combination of clinical, laboratory tests, radiology, endoscopy, and pathology aspects. Diagnosis, evaluation of severity, remission flare up and prognosis still challenge the clinicians. Laboratory biomarkers are noninvasive or microinvasive indices which are more rapid and cost less compared to other techniques, thus relieving physiological and financial burden for patients (3). The incidence and prevalence of IBD are increasing all over the world (4, 5). Up to 20% of affected persons have a history of IBD in their family, with the highest risk among first-degree relatives (6). In recent years, most progress has been made in understanding the genetic contribution to pathogenesis of IBD and other immune mediated diseases, with more than 75% of overlap with genetic risk loci (7).

Both CD and UC are complex diseases genetically, in which hundreds of independent genetic loci contribute to disease susceptibility (8). Nevertheless, the molecular mechanisms and functions of many IBD associated genes remain unknown. In addition to genetics, IBDs are powerfully influenced by microbiological and environmental aspects. The influence of environment on IBDs is best illustrated by the unreasonably high prevalence of disease in developed countries, and prominent rates of disease among identical twins discordantly (about 50% for CD and 10% for UC). In one way, better understanding of the involvement of inflammatory mechanisms combined with a lack of sufficient response to conventional medical therapy, has permitted cytokines and biomarkers as biologic targets to appear as an important modality in IBD diagnosis, prognosis and treatment (9). Hence, a better understanding of the biomarkers’ mechanisms and the immune response in IBD may give rise to new alternative approaches for early diagnosis and prognosis in the follow-up process of patients. This review will address noninvasive biomarkers as a personalized panel in IBD management.

**1-Biomarkers**

Biomarkers are defined as measureable substances derived from a tissue or biofluid specimens. This broad description can include common laboratory indices, such as hemoglobin concentration, or even biological techniques and expression of gene profile (blood cells ‘samples). They are becoming particularly important as less-invasive, resource-benefit and cost-effective modalities for a curative approach in IBD (3). Numerous genes have been linked to IBD, and many of these can be associated with alterations in immune pathways. Studying the interactions with various constituents and elements such as biomarkers and cytokines in the immunoregulatory pathways concerned in IBD will definitely foster new modalities for diagnosis, prognosis, and treatment panels in IBD patients. Recent studies have focused on more than 163 gene loci related to IBD, of which at least 110 are associated with both UC and CD (8). Currently, many biomarkers are extensively used in clinic, but none of them can be used separately for accurate diagnosis of IBD, or to subclassify IBD patients into CD and UC. Overall, they can only be used as an adjunct to procedure evaluation or in disease monitoring (10). Besides clinical and endoscopic scores, serological and immunologic biomarkers are broadly used to evaluate disease process. In comparison to invasive procedures, such biomarkers have the advantage of being safe. Nonetheless, they do not always correlate with the presence of endoscopic lesions (10, 11). Biomarkers show low sensitivity in mild to moderate IBD patients. Although histologic improvement of the intestine is a good predictor of disease outcome (12, 13), relying only on this measure to control response to treatment during early stage may yield low sensitivity. In a few patients, histologic response may be late and represent only the delayed stages of recovery.

**2-Serologic biomarkers**

 Serum antibodies against microorganism, auto or foreign antigen, either alone or in combination, have been recognized as possible parameters for diagnosing and differentially diagnosing between CD and UC. Many meta-analyses and systematic reviews provide the data on the results of studies conducted over the past 20 years to assess the clinical usefulness of serological and immunological biomarkers (14-17). 

CRP: C- reactive protein and Hypersensitive C- reactive protein. C-reactive protein (CRP) is recognized as one of the most important proteins in acute inflammation phase (18). CRP may not be used alone as a diagnostic or treatment biomarker in CD and UC because it has insufficient functional characteristics to act as a substitute for clinical endoscopic or radiographic findings. CRP is a noninvasive adjunctive index that can be used in both CD and UC to direct the evaluation by further endoscopy or radiography. Breakdown of serum CRP level following therapy initiation should prompt further endoscopic and/or radiographic evaluation, irrespective of symptoms in CD. Elevated serum CRP levels at baseline suggest response to antitumor necrosis factor (TNF) agents, and normal serum CRP level is modestly associated with clinical and endoscopic remission. Hemoglobin and CRP levels are used to help anticipate clinical relapse in the situation of withdrawal of therapeutic phase. In UC, the value of CRP as an adjunctive guide is greatest for disease severity, in which constant elevation, in addition to low albumin levels, is associated with the need for colectomy. Normal serum CRP level is associated with clinical remission and mucosal healing (19). In CD, there is evidence that extra-enteric inflammation based on enterography and computed tomography correlates with CRP (20), and CRP is more often elevated in individuals with colonic involvement. There is a modest correlation between endoscopic disease activity and CRP levels in CD (11, 21). In addition to biologic trials, thiopurine trials have confirmed CRP as a key biomarker for prognostic approach of relapse. Two studies exploring thiopurine withdrawal (22, 23) demonstrated the elevated CRP level to be an independent predictor of relapse after withdrawal of azathioprine, in addition to a low hemoglobin level. Among patients treated with combination therapy, in whom infliximab is discontinued, CRP is also a predictor of relapse (24). Only some randomized clinical trials (RCT) assessing therapy in active phase of UC have used CRP as an end point. An early multicenter prospective RCT planned to assess the efficacy, tolerability and safety of treatment of IBD using CRP as a secondary end point (25).

ASCA and ANCA: Antineutrophil Cytoplasmic Antibodies. Antineutrophil cytoplasmic antibodies (ANCAs) are antibodies for granules of neutrophil cytoplasm, which were first reported in UC patients in 1990 (26). Atypical perinuclear ANCA (pANCA) is DNase sensitive (27); it increases considerably in UC (28). Follow-up of a study with prospective recruiting of IBD unclassified (IBD-U) cases demonstrated that 64% of patients with UC were anti-Saccharomyces cerevisiae antibody (ASCA)−/pANCA+ (29). Another population-based survey described that the positive rate of pANCA is 55% in UC, 48% in rheumatoid arthritis, and 32% in healthy people (30). Dotan and coworkers studied 152 UC patients, 54 CD patients, and 60 IBD-U, reporting that the specificity and sensitivity of pANCA are 96.3% and 43.3% separately compared to the healthy control group (31). The earliest described and best characterized serologi¬cal biomarkers for IBD are anti-Saccharomyces cervisiae anti¬body (ASCA) and atypical perinuclear anti-neutrophil cytoplasmic antibody (pANCA). ASCA is chiefly linked with CD and pANCA with UC (26, 32, 33). Apart from pANCAs and ASCAs, the other serological antibodies such as anti-OmpC, ALCAs, ACCAs, AMCAs, anti- L, and anti-C and pancreatic autoantibodies (PAB) also supply to diagnosis of IBD and differential diagnosis of other diseases (14, 16, 34, 35). Antibodies to microbial constituents have been associated with CD; these include antibodies to Escherichia coli outer membrane porin (OmpC), Pseudomonas fluorescens-associated sequence (15), and bacterial flagellin (30, 36-40). A number of additional anti-glycan antibodies have also been associated with CD: anti-mannobioside carbohydrate antibody (AMCA), anti-laminaribiodise carbohydrate antibody (ALCA), anti-chitobioside carbohydrate antibody (ACCA), anti-laminarin (Anti L), and anti-chitin (Anti C) (22). Separately or in combination, serological biomarkers display high specificity but less sensitivity for correct diagnosis of IBD patients, thus having a restricted value for disease screening.

When trying to differentiate UC from CD, overall, the combination of ASCA and pANCA yields the best specificity (>90%) and sensitivity (~55%), but has low sensitivity (~35%) when trying to differentiate colonic UC from CD (14, 32, 41). Adding OmpC, I2, and CBir1 antibod¬ies seems to add a small benefit in these situations (35, 42). Combining several anti-glycan antibodies with ASCA and pANCA increases sensitivity at the cost of specificity (31).

Attempts to recover the diagnostic usefulness of serological biomarkers for pANCA and antimicrobial antibodies have joined them in a panel with four gene variants (ATG16L1, rs2241880; NKX2 3, rs10883365; ECM1, rs3737240; and STAT3, rs744166), five inflammatory markers (C-reactive protein (CRP), serum amyloid A (SAA), intracellular adhesion molecule 1 (ICAM), vascular cell adhesion mol¬ecule 1 (VCAM), and vascular endothelial growth factor (VEGF)), and two additional antimicrobial serological markers (A4-Fla2 and FlaX) (43). With this combination, the area under the receiver operating curve (AUROC) to discriminate IBD from non-IBD increases to 0.87 (95% CI –0.04 to 0.04), from 0.80 (95% CI –0.05 to 0.05) with a panel of seven serological markers (P <0.001). The mixture panel of biomarkers was also better at distinguishing between CD and UC among patients with known diagnoses compared with either a previous panel of only serological markers (AUROC: 0.93 (95% CI –0.04 to 0.04) versus 0.78 (95% CI –0.06 to 0.06); P <0.001) or any separate marker alone. Importantly, it should be noted that the utility of this test must still be confirmed in a prospective cohort and in populations of patients for whom the diagnosis is unclassified. Because of ethnic variances in antibody prevalence, these panels also need to be assessed in populations not derived from Northern Europe to determine their clinical utility beyond the populations in which they have been developed (14). So, at best, information about serological markers should be used as adjuncts to conventional methods of diagnosing and differentiating IBD in patients of Northern European descent. 

ESR: Erythrocyte Sedimentation Rate. Largely similar to CRP, it is a nonspecific index of systemic inflammation, which can be raised in a number of conditions and therefore, not specific to IBD. One study looking at the correlation between CRP and ESR and endoscopic activity in patients with UC found only a very modest correlation. Endoscopic activity was better correlated with CRP than with ESR (14).

PLT: Platelet Count. Platelets (PLT) increase in patients with IBD, which contribute to high-coagulated state of IBD, such as the formation of microthrombus (44-46). Moreover, reticulated platelet levels increase significantly in patients with UC (47).

MPV: Mean Platelet Volume. Mean platelet volume (MPV) indicates the average size of platelets; it could reflect the rate of platelet stimulation and production. Kapsoritakis et al (48) found out that MPV decreased significantly in active-IBD, and it is negatively correlated with some markers of inflammation, such as white blood cell, CRP, and ESR. However, another investigation did not show any relationship between this parameter and disease activity (49). The decline of MPV may be related with disturbance of thrombus formation when inflammation occurs (48).

**3-Genetic predisposing markers**

Much progress has been made in detection of gene variants in patients with IBD through genome-wide association studies. The first study of this type described a presumed CD susceptibility locus on chromosome 16, which contains the NOD2 coding gene (50, 51). A recent meta-analysis of CD and UC genome-wide association studies reported on findings from more than 75,000 cases and controls. The authors identified 71 new associations increasing the total number of confirmed IBD susceptibility loci up to 163 (51). They found that most loci contributed to both phenotypes (52). The IBD chip European plan assessed a number of CD single nucleotide polymorphisms (SNP). The NOD2 gene was found to be the most important genetic factor. It was shown to be an independent predictive factor for ileal location, stenosing, and penetrating CD and was also associated with a more complicated disease course and the need for surgery (53). A pediatric study of 169 patients with IBD identified two patients with very early onset UC to have rare deleterious variations of NOD2, as well as potentially pathogenic variations in the BACH2 and IL-10 genes (54). Ellinghaus et al (55) found that variants in two genes, PRDM1 and NDP52, determined susceptibility to CD. PRDM1 was found adjacent to a CD interval identified in genome-wide association studies. PRDM1 encodes PR domain-containing 1, also known as B-lymphocyte–induced maturation protein (Blimp-1), and is involved in regulation of terminal B- and T-cell differentiation (56). NDP52 (nuclear domain 10 protein 52) is known as a cytosolic protein related to selective autophagy of intracellular bacteria and signaling molecules (57). Mutations of IL-10 receptors have been found in IBD. Three homozygous mutations in IL10RA and IL10RB were identified from 4 of 9 patients with early onset colitis. The mutations disabled IL-10–mediated signaling, leading to onset of disease as early as 3 months old. Stem cell transplantation resulted in remission of disease. IL-102/2 mice have been demonstrated to spontaneously develop colitis illustrating the powerful immunosuppressive effect of IL10 (58). Sequencing analysis of a 15-month-old male child with CD-like illness identified a mutation on the X chromosome in the X-linked inhibitor of apoptosis (XIAP) gene (58). This was the first time the XIAP protein had been implicated in CD. 

Several common variants in the IL-23 receptor gene (IL23R) are reported to be clearly associated with both CD and UC susceptibility (59). But in some part of East Asia, IL23R variants do not show any relation with CD (60-62). IL23R is a CD susceptibility gene, but different IL23R variants are likely to carry variable disease-modifying effects in different populations.

The gene also affects the strategies of treatment. A research in Germany showed that homozygous carriers of IBD risk-increasing IL23R variants were more apt to respond to anti-TNF than homozygous carriers of IBD risk-decreasing IL23R variants (63).

MicroRNAs (miRNAs) are single-stranded noncoding RNAs, around 22 nucleotides in length, that have remained highly conserved throughout evolution (64). Since they were first described in the 1990s, more than 1600 miRNAs have been described in humans. miRNAs regulate gene expression and thus, a number of biological processes such as cell proliferation, differentiation, and death. Changes in miRNAs expression have been associated with a number of diseases including IBD (65, 66). One group reported for the first time that miRNAs were differentially expressed in the colonic mucosa of UC and then went on to demonstrate that peripheral blood miRNAs can distinguish active IBD subtypes from each other and from healthy controls and also that distinctive differences in miRNA expression in the distal ileum and colon in patients with CD (67, 68). With regard to monitoring of disease, miRNA levels have been shown to vary between active and inactive Crohn’s and UC in both colonic tissue and serum samples (68). Another study found that MiR-31 expression levels increased with disease progression and accurately discriminated neoplastic from normal or chronically inflamed tissues in patients with IBD (69). There is also some promise in the utilization of miRNAs in assessing response to treatment. miRNA levels have been shown to be significantly different before and after glucocorticoid therapy (70). Another group looked at serum samples of patients receiving infliximab-induction therapy; miRNA levels of let-7d and let-7e were significantly increased in the remission group (71).

**4-Immunological biomarkers**

Investigation of the immunologic aspect of IBD has been dominated for a long time by studies of mucosal immunity, especially the T cell response. Available evidence suggests that the dysfunctions of innate and adaptive immune pathways contribute to the aberrant intestinal inflammatory response in patients with IBD (72, 73). Nonetheless, several immunoregulatory mediators are upregulated in the intestinal mucosa of patients with IBD (74, 75) The innate immune response represents our first line of defense against pathogens (76, 77) This form of immunity is initiated by recognition of microbial antigens, which is mediated by pattern recognition receptors including tolllike receptors (TLRs) on the cell surface and NOD-like receptors in the cytoplasm (78). Most studies in the last two decades have focused on the role of abnormal adaptive immune responses in the pathogenesis of IBD. Studying the interactions between various elements and constituents (T cells and cytokines) of the immunoregulatory pathways involved in IBD will certainly open new horizons for intent to treat in IBD patients. Recent advances lead to the understanding of the development and function of regulatory T cells (Treg), and regulatory cytokines that predict the prognosis of IBD (2).

**5-Regulatory T cells**

Regulatory T cells (Tregs) are critical suppressors of autoimmunity and enteropathies in mice and humans. Tregs come in two flavors; the CD4+Foxp3+ Tregs have been called “naturally occurring” Tregs that develop in the thymus “suppressor” T-cell populations that are generated in vitro. Suppressor Tregs differentiate from mature naive T cells in parallel with conventional effector subsets (i.e., Th1, Th2, Th17) in peripheral lymphoid organs (79). Both natural Tregs and suppressor Tregs influence the bystander activation of T cells—and thus contribute to immune tolerance—in a manner that requires FOXP3, a forehead box transcription factor that is mutated in the autoimmune Scurfy mouse and in human immune dysregulation, polyendocrinopathy, enteropathy, and X-linked (IPEX) patients (80). FOXP3 can be used as a good marker for mouse CD4+CD25+ T cells although recent studies have also shown evidence for FOXP3 expression in CD4+CD252 T cells. In humans, FOXP33 is also expressed by recently activated conventional T cells and thus does not specifically identify human Tregs (2). Although many data clearly implicate Tregs in control of mucosal inflammation, whether or not perturbed Treg function is a common feature of IBD remains controversial. Some studies have reported reduced Treg numbers in inflamed mucosal biopsies from IBD patients (80). Although a unifying mechanism of Treg-mediated immune suppression is not known, several molecules have been implicated in the regulation of Treg-dependent intestinal homeostasis (79). Chief among these is IL-10; Treg-specific ablation of Il10 is sufficient to induce spontaneous colitis (81). Polymorphisms in genes encoding for subunits of the IL-10 receptor (IL10RA, IL10RB) are also associated with human IBD, particularly very early onset colitis (80). Tregs also express IL-35, a relatively recent addition to the cytokine family. Like most cytokines, IL-35 is a heterodimeric protein, consisting of EBI3 (encodes for IL-27b) and IL12A (encodes for IL-12p35) (82). Two additional epithelial-derived cytokines, namely IL-18 and the “alarming” IL-33, have been recently suggested to enforce Treg-mediated control of colonic inflammation. Notably, these cytokines are unique from other Treg-related pathways that systemically boost Treg development and/or function (e.g., TGF-b, IL-10, IL-35, CTLA-4, etc), in that IL-18–driven or IL-33–driven Treg function seems to have tissue specificity for the gut (83-85). 

**6-Cytokines**

Cytokines, small proteins produced by immune cells, facilitate communication between cells and have essential functions in cell development and differentiation. The pro-inflammatory cytokines play a critical role in the immune response of IBD; however, regulation of these cytokines is generally mediated by the immunoregulatory cytokine (2). There are several processes or pathways in disease pathogenesis. These include regulation of intestinal barrier function and antimicrobial activity, endoplasmic reticulum stress, autophagy, and CD4+ T helper (Th) cell development and function. Each pathway interacts with the others to coordinate intestinal homeostasis in healthy individuals and to confer risk in IBD patients. Cytokines regulate each of these pathways, thus enabling molecular crosstalk between the epithelium, mucosal immune cells, and the microbiota. Accordingly, cytokines, cytokine receptors, and regulators of cytokine signaling are among the most over-represented class of genes associated with IBD (52). 

**6-1-Interleukin-10**

In 2001, six immune mediators (IL-10, IL-19, IL-20, IL-22, IL-24, and IL-26) were grouped into the so-called IL-10 family of cytokines. The IL-10 family cytokine is essential for maintaining the integrity and homeostasis of tissue epithelial layers. Members of this family can promote innate immune responses from tissue epithelia to limit the damage caused by viral and bacterial infections. These cytokines can also facilitate the tissue-healing process in injuries caused by inflammation (86, 87). Mutations of IL-10 receptors have been found in IBD. Three homozygous mutations in IL10RA and IL10RB were identified in four out of nine patients with early onset colitis. The mutations disabled IL-10–mediated signaling, leading to onset of disease as early as 3 months old. Stem cell transplantation resulted in remission of disease. IL-102/2 mice have been demonstrated to spontaneously develop colitis illustrating the powerful immunosuppressive effect of IL10 (88).

**6-2-Interleukin-19**

The IL-19 was discovered in 2000 and belongs to the super family of IL-10. The IL-19 is primarily produced by keratinocytes, epithelial cells, monocytes, and B cells, and its expression can be induced by lipopolysaccharide, granulocyte–macrophage colony-stimulating factor, IL-6, and tumor necrosis factor. Monocyte IL-19 production is downregulated by interferon gamma (89-91). Azuma et al demonstrated that IL-19 regulates the inflammatory process in acute and chronic conditions as well as inducing the mucosa healing of the intestinal epithelium in a murine model of IBD (92). Yamamoto-Furusho et al found that IL-19 polymorphisms (rs2243188 and rs2243193) might have a protective role in the development of UC in Mexican individuals (93).

**6-3-Interleukin-22**

IL-22 is a member of the IL-10 subfamily, which was identified as T-cell–derived cytokine. Expression of IL-22 is induced in several human inflammatory conditions including IBD (94). Sugimoto et al (95) found that IL-22 gene delivery led to rapid amelioration of local intestinal inflammation in a dextran sulfate sodium–induced model of acute colitis. Expression of the IL-22 receptor is restricted to innate immune cells. Findings have confirmed the potential role of the IL-22 gene in the pathogenesis of UC and suggest that IL-22 might be involved as a defense mechanism by enhanced mucus production (96).

**6-4-Interleukin-12**

IL-12 cytokine family includes IL-12, IL-23, IL-27 and its most recent member, IL-35. Each cytokine is formed of two subunits, some of which form bioactive cytokines themselves, and others which are shared among family members. This characteristic provides these cytokines with a great degree of interaction potential not present in other cytokine families, both in the context of receptor binding and other molecular interactions. This diversity is perhaps responsible for the highly variable biological functions that have been reported in the context of preclinical studies of inflammation (97) Expression of IL-12 and IL-23 are highly up-regulated in patients with the inflammatory bowel disease (98).

**6-5-Interleukin-27**

IL-27 is a heterodimeric type I cytokine in the IL-12 cytokine family. IL-27 expression induced by TLR signaling has been shown to require interferon-a in human macrophages and interferon regulatory factor 3 in murine dendritic cells (99, 100). IL-27 boasts a diverse repertoire of functions in both the adaptive and innate branches of the immune system; however, the most well-known and thoroughly investigated functions of IL-27 relate to its ability to regulate the adaptive immune system by modulating T cell function. After identifying the receptor responsible for IL-27 signaling, Pflanz et al showed that IL-27 induced proinflammatory gene expression in both human mast cells and monocytes (101). Both subunits of the IL-27 receptor, IL-27Ra and gp130, are expressed at low levels in normal intestinal epithelial cells, but are up-regulated in inflammation in both epithelial cells and infiltrating leukocytes (102). It has not been definitively determined whether IL-27 ameliorates or promotes intestinal inflammation, because seemingly contradictory roles for IL-27 have been reported in murine models of IBD (103). Increased expression of IL-27 in inflamed segments of the intestinal mucosa has been demonstrated in both Crohn’s disease and ulcerative colitis (102, 104). Patients with active Crohn’s disease have also been shown to have both significantly increased serum IL-27 and soluble IL-27Ra relative to healthy controls; however, despite an overall positive correlation between these two values, the ratio of cytokine to soluble receptor varied widely among patients (105).

**6-6-Interleukin-35**

IL-35 is the newest member of the IL-12 family, and it has anti-inflammatory/immunosuppressive properties. The IL-35 is constituted by heterodimerization of two subunits Epstein–Barr virus–induced gene 3 (EBI3) and the IL-12 p35 subunit (IL-12A) (105). Li and colleagues showed that the IL-35 is not constitutively expressed in non-stimulated human tissues; IL-35 is produced by Tregs (Foxp3+ Tregs) and by activated dendritic cells, (106) and this novel cytokine can down-regulate Th17 cell development and inhibit autoimmune inflammation (107) (108). The increased immunity found in mice lacking the IL-35 production by B cells was associated with a higher activation of macrophages and inflammatory T cells, as well as an increased function of B cells as antigen-presenting cells. Moreover, Wirtz et al demonstrated that IL-35 protects against development of T-cell–dependent colitis in mice (109).

**6-7-Interleukin-24**

IL-24 is a member of the IL-10 family of cytokines. IL-24 was originally identified as a tumor suppressor molecule (melanoma differentiation associated gene 7). IL-24 shares between 20% and 30% amino acid homology with IL-10, IL-20, and IL-22. The human IL-24 has been reported to induce the expression of proinflammatory cytokines such as tumor necrosis factor alpha and IL-6 in monocytes (110). In vivo, IL-24 is predominantly expressed by skin tissue cells during inflammatory conditions such as psoriasis (111). The IL-24 acts on colonic epithelial cells to elicit JAK1/STAT3 activation and the expression of mucins, supporting its suppressive effects on mucosal inflammation in IBD (112). The gene expression of IL-24 was also significantly increased in the group of active UC as compared with the UC remission group. Therefore, IL-24 is expressed in infiltrating immune cells and epithelial cells. Importantly, the number of IL-24–expressing cells in the inflamed colonic tissue from patients with UC was higher than non-inflamed tissue. These findings confirm the role of IL-24 in the pathogenesis of UC (113).

**Table.1:** Cytokines’ functions in IBD

**Cytokine**	**source**	**function**
**IL-10**	Treg	Inhibits proinflammatory cytokine expression by innate/adaptive immune cells; STAT3-dependent signaling
**IL-12**	Th1	Stimulates Th1 differentiation and effector cytokine production (IFNg, TNF-a); STAT4-dependent signaling
**IL-19**	CD4+ T cells, CD8+ T cells	IL-19 drives pathogenic innate immune responses in the colon
**IL-20**	T cells, maturing DCs	IL-20 is crucial in immune responses, regulation of inflammation, hemopoiesis, and epidermal cell. IL-20 may play a pathophysiological role in the Th2 immune response
**IL-21**	Tfh	Growth/differentiation factor for germinal center B cells; signals through STAT1/STAT3
**IL-22**	DCs, NK cells, neutrophils Th17, Th22	Enforces epithelial barrier function; stimulates expression of antimicrobial peptides, defensins; signals through STAT3
**IL-23**	Th17	Activates proliferation and cytokine expression in pathogenic Th17 cells; STAT3-dependent signaling
**IL-24**	peripheral B cells, CD4+T CD8+ cells,	The human IL-24 has been reported to induce the expression of proinflammatory cytokines such as tumor necrosis factor alpha and IL-6 in monocytes
**IL-4**	Th2	Induces IgE class-switch recombination in B cells; induces Th2 differentiation and IL-13 expression; signals through STAT6
**IL-12**	Th1	Stimulates Th1 differentiation and effector cytokine production (IFNg, TNF-a); STAT4-dependent signaling
**IL-13**	Th2	Impairs epithelial barrier function; promotes mucosal fibrosis through induction of TGFb1 expression; signals through STAT6
**IL-5**	Th2	Growth/differentiation factor for B cells, eosinophils; STAT1/STAT3/ STAT5-dependent signaling
**IL-35**	Tregs	IL-35 downregulates Th17 cell development and inhibits autoimmune inflammation. IL-35 is required for the suppressive function of Treg in vitro and in vivo. IL-35 protects against development of T-cell–dependent colitis in mice
**IL-37**	Tregs	IL-37 is a fundamental inhibitor of innate immunity
**IL-17A**	Th17	Enforces epithelial barrier function; induces proinflammatory cytokine/ chemokine expression in endothelial cells; NF-kB–dependent signaling
**IL-9**	CD4+ T cells, CD8+ T cells	Impairs mucosal wound healing; regulates epithelial cell proliferation, barrier function; STAT3/STAT1/MAPK dependent signaling
**TGF-b1**	Treg	Regulates differentiation of iTreg, nonpathogenic Th17 cells; signals via SMAD transcription factors
**IFNg**	Th1	Activates MF, NK cells, CD8+ T cells; increases MHCII expression; STAT1-dependent signaling
**TNF-a**	Th1,Th2,Th17	Regulates immune cell survival and function; induction of apoptosis, cachexia, and inflammation; signals through NF-kB and MAPK pathways
**IL-18**		
**IL-26**	Monocytes ,memory T cells	stimulation increases the mRNA expression of proinflammatory cytokines but decreases cell proliferation.

**6-8-Interleukin-37**

IL-37 is an anti-inflammatory cytokine in the IL-1 ligand family (114). The IL-37 plays an important role in the development and progression of inflammatory and autoimmune diseases (115); it may be associated with the development of pediatric IBD (116). IL-37, which is normally expressed at low levels in peripheral blood mononuclear cells, mainly monocytes, and dendritic cells (DCs), is rapidly up-regulated in the inflammatory context (117), and therefore, IL-37 

conversely inhibits the production of inflammatory cytokines in peripheral blood mononuclear cells and DCs of patients with systemic lupus erythematosus.

In addition, IL-37 effectively suppresses the activation of macrophage and DCs. DCs-expressing IL-37 are tolerogenic, thereby impairing activation of effector T-cell responses and inducing Treg cells. The IL-37 thus emerges as an inhibitor of adaptive immunity (118).

These cytokine networks have emerged as key players in the development and function of Tregs that may be leveraged clinically to improve IBD therapy. In addition, the universe of CD4+ Th cell subsets continues to expand. Given their relatively recent discovery, far less is known about the role of these “emerging” Th cell subsets in IBD, although at least two of these merit brief discussion, namely Th9 and follicular helper T (Tfh) cells (80). Table 1 presents cytokines’ functions in IBD.

## Conclusion

In recent years, progress in clinical, laboratory and immunopathology research has led to better understanding of the role of inflammatory mediators in the pathogenesis of IBD. The development of noninvasive biomarkers, which are acceptable and well taken up by patients, would be the ideal (58). Cost-effectiveness issues also need to be taken into account. Many studies have touched on current tests including CRP and ESR, which are cheap and reliable, but hugely nonspecific. FCP, now a very widely used tool, has allowed physicians to take steps toward more accurate diagnosis and predict the prognosis of IBD patients. However, the more novel biomarkers, including genetic testing, miRNAs, and metabolomics may in the future lead to highly accurate testing, not only in diagnosis of symptomatic patients but also potentially in screening of susceptible individuals before disease onset (67). Studies have also reported the development of biomarkers enabling assessment of response to treatment, as in the case of anti-TNFa treatment (58, 119). It is believed that an altered balance between regulatory and inflammatory cytokines contributes to perpetuate the mucosal inflammation in both CD and UC. Since there is evidence that the tissue damaging immune response is driven by multiple cytokines related to inflammatory pathways, it is logical to hypothesize that simultaneously targeting two or more of these signals could be more advantageous than just selective single pathways for early diagnosis and also predicting the prognosis of IBD (72, 120). Various approaches have been developed for inhibiting such pathways and are ready to go into the clinic for treatment. However, in designing clinical interventions around these new drugs, it should be taken into consideration that inhibition of inflammatory cytokines could be associated with severe side-effects, as these molecules are also involved in the regulation of physiological processes and immune responses against infections and neoplasias (79). Another promising therapeutic strategy is to restore counter-regulatory mechanisms which are defective in IBD. Since it is conceivable that no drug fits all patients, further experiments will be necessary to identify biomarkers that predict disease prognosis and also responsiveness to the anti-cytokine based therapy as well as to ascertain which biological therapy will be most effective for the individual patient.

The management of IBD requires a personalized approach to early onset diagnosis, prognosis and treatment since a heterogeneous group of patients in subcategories of disease have inherently variable courses.

Currently, genetic, serologic, and immunologic biomarkers have been applied in practice and many of them still need to be confirmed in large cohort and clinical research. An accurate panel of biomarkers leads to personalize approach for predicting prognosis and also response to therapy. There is hope that the ultimate development of a biomarker signature may yield significant advances in our ability to identify those patients with the greatest risk for severe disease, who would thus benefit most from aggressive and individualized therapies.

## Conflict of interests

 The authors declare that they have no conflict of interests.
